# Detection of *Plasmodium simium* gametocytes in non-human primates from the Brazilian Atlantic Forest

**DOI:** 10.1186/s12936-023-04601-7

**Published:** 2023-06-02

**Authors:** Lara Cotta Amaral, Yanka Evellyn Alves Rodrigues Salazar, Denise Anete Madureira de Alvarenga, Anielle de Pina-Costa, Ana Júlia Dutra Nunes, Júlio Cesar de Souza Junior, Gustavo Henrique Pereira Gonçalves, Zelinda Maria Braga Hirano, Sílvia Bahadian Moreira, Alcides Pissinatti, Cláudio Tadeu Daniel-Ribeiro, Taís Nóbrega de Sousa, Cristiana Ferreira Alves de Brito

**Affiliations:** 1 Grupo de Pesquisa em Biologia Molecular e Imunologia da Malária, Instituto René Rachou, Fundação Oswaldo Cruz (Fiocruz), Belo Horizonte, Brazil; 2 Laboratório de Doenças Febris Agudas, Instituto Nacional de Infectologia Evandro Chagas, Fiocruz, Rio de Janeiro, Brazil; 3 Centro de Pesquisa, Diagnóstico e Treinamento em Malária, Fiocruz, Rio de Janeiro, Brazil; 4 Laboratório de Pesquisa em Malária, Instituto Oswaldo Cruz, Fiocruz, Rio de Janeiro, Brazil; 5 Departamento de Doenças Infecciosas e Parasitárias, Escola de Enfermagem Aurora de Afonso Costa, Universidade Federal Fluminense, Niterói, Brazil; 6 Programa de Conservação do Bugio Ruivo, Joinville, Brazil; 7 Centro de Pesquisas Biológicas de Indaial, Indaial, Brazil; 8 Universidade Regional de Blumenau – FURB, Blumenau, Brazil; 9 Centro de Primatologia do Rio de Janeiro/INEA, Guapimirim, Brazil; 10 Centro Universitário Serra dos Órgãos, Teresópolis, Brazil

**Keywords:** Malaria, Zoonoses, Infectious disease transmission, *Plasmodium simium*, Non-human primate, Gametocytes

## Abstract

**Background:**

*Plasmodium* species of non-human primates (NHP) are of great interest because they can naturally infect humans. *Plasmodium simium*, a parasite restricted to the Brazilian Atlantic Forest, was recently shown to cause a zoonotic outbreak in the state of Rio de Janeiro. The potential of NHP to act as reservoirs of *Plasmodium* infection presents a challenge for malaria elimination, as NHP will contribute to the persistence of the parasite. The aim of the current study was to identify and quantify gametocytes in NHP naturally-infected by *P. simium*.

**Methods:**

Whole blood samples from 35 NHP were used in quantitative reverse transcription PCR (RT-qPCR) assays targeting *18S rRNA*, *Pss25* and *Pss48/45* malaria parasite transcripts. Absolute quantification was performed in positive samples for *18S rRNA* and *Pss25* targets. Linear regression was used to compare the quantification cycle (Cq) and the Spearman's rank correlation coefficient was used to assess the correlation between the copy numbers of *18S rRNA* and *Pss25* transcripts. The number of gametocytes/µL was calculated by applying a conversion factor of 4.17 *Pss25* transcript copies per gametocyte.

**Results:**

Overall, 87.5% of the 26 samples, previously diagnosed as *P. simium,* were positive for *18S rRNA* transcript amplification, of which 13 samples (62%) were positive for *Pss25* transcript amplification and 7 samples (54%) were also positive for *Pss48/45 *transcript. A strong positive correlation was identified between the Cq of the *18S rRNA* and *Pss25* and between the *Pss25* and *Pss48/45* transcripts. The *18S rRNA* and *Pss25* transcripts had an average of 1665.88 and 3.07 copies/µL, respectively. A positive correlation was observed between the copy number of *Pss25* and *18S rRNA* transcripts. Almost all gametocyte carriers exhibited low numbers of gametocytes (< 1/µL), with only one howler monkey having 5.8 gametocytes/µL.

**Conclusions:**

For the first time, a molecular detection of *P. simium* gametocytes in the blood of naturally-infected brown howler monkeys (*Alouatta guariba clamitans*) was reported here, providing evidence that they are likely to be infectious and transmit *P. simium* infection, and, therefore, may act as a reservoir of malaria infection for humans in the Brazilian Atlantic Forest.

**Supplementary Information:**

The online version contains supplementary material available at 10.1186/s12936-023-04601-7.

## Background

According to the World Health Organization, in 2021 there were 247 million cases and 619,000 deaths from malaria in 85 endemic countries [1]. In Brazil, 145,188 malaria cases were reported in 2020, showing a reduction of 7.8% compared to the previous year [2]. This reduction reflects the efforts which have been made to eliminate malaria in Brazil through the implementation of the “National Malaria Elimination Plan”, which aims to reduce the number of autochthonous cases to less than 68,000 by 2025, the number of deaths to zero by 2030, and to eliminate the disease from Brazilian territory by 2035. However, there are many challenges to achieving these goals, including the emergence of parasites resistant to anti-malarial drugs, the need for better vector control strategies, human migration, the need for effective surveillance and tools to identify foci of infection in low transmission areas, and the high prevalence of asymptomatic and submicroscopic infections, which can only be detected by molecular techniques [3].

Most human malaria infections are caused by five *Plasmodium* species: *Plasmodium falciparum*, *Plasmodium vivax*, *Plasmodium malariae*, *Plasmodium ovale* and *Plasmodium knowlesi. Plasmodium knowlesi* is a primate malaria parasite that primarily affects long-tailed and pig-tailed macaques, which has emerged as an important human pathogen in recent years, responsible for over 70% of human cases in southeastern Asia [4–7]. *Plasmodium* species that cause infection in non-human primates (NHP) are of great interest because they can be naturally-transmitted to humans, thus representing a challenge to malaria elimination.

Besides *P. knowlesi*, at least other three species, which infect primarily NHP, are involved in the zoonotic transmission of malaria to humans, including *Plasmodium cynomolgi* in Asia [8] and *Plasmodium brasilianum* and *Plasmodium simium* in the Americas [9–12]. *Plasmodium simium*, a parasite of a small number of species of Neotropical monkeys, is restricted to the Atlantic Forest from Southeast and South regions of Brazil and it was recently shown to cause zoonotic infections in humans [12]. An outbreak attributed to *P. vivax* in the Atlantic Forest areas of the state of Rio de Janeiro have been shown to be, in fact, caused by *P. simium* [12]. *Plasmodium simium* and *P. vivax* are genetically, morphologically, and immunologically similar, and the brown howler monkey (*Alouatta guariba clamitans*) has been suggested to be the main reservoir host of *P. simium* [9, 13, 14]. However, the reservoir status of howler monkeys is uncertain, since the only evidence currently available from the field is that *P. simium* infection occurs in this species, but their potential for natural transmission (i.e. infectivity to mosquitoes) in this setting is not yet known.

Despite the importance of parasites with potential for zoonotic transmission, little is known about their epidemiological importance to infect and cause disease in humans. The potential for NHP to act as reservoirs of *Plasmodium* infection for humans presents a challenge for malaria elimination, as they contribute to the persistence of the parasite and act as source of re-introduction into human populations, in areas where infection has otherwise controlled [15–17]. In this context, the occurrence and density of gametocytes, which are the infective stages of *Plasmodium* for mosquitoes, in reservoir hosts are important factors to estimate their potential for malaria transmission [18–20]. Furthermore, this information could be used for modelling the dynamics of zoonotic malaria transmission [16, 21].

Among the proteins expressed in large quantities after gametocyte activation in the midgut of the mosquito are the P25 orthologues Pfs25 and Pvs25, of *P. falciparum* and *P. vivax*, respectively [22]. Although the P25 protein is expressed on the surface of malaria parasite stages occurring within the mosquito, the transcription of its gene begins within, and - of the vertebrate host erythrocytic stages - is specific to female gametocytes. The *Pvs25* gene is highly conserved among *P. vivax* isolates [23–25], making it a useful molecular marker for detection of sexual stage malaria parasites [16]. Another possible stage-specific target for monitoring the sexual stages of *Plasmodium* is the P48/45 protein, which is expressed in both male and female sexual stages starting at stage II gametocytes and continuing until fertilization is complete and forms a complex with P230 [26–28]. Despite information scarcity in the literature, it is known that P48/45 is involved in male gamete fertility, but not female fertility [29], and is evolutionarily conserved among *Plasmodium* species, again exhibiting low levels of genetic diversity [30]. A transcriptional study of gametocyte genes from *P. vivax* showed that they cluster in two groups of co-regulated genes, one includes Pvs25 and the other Pvs48/45, suggesting that the regulation of male and female genes is independent of each other [31].

The aim of the current study was to assess the potential for malaria transmission by naturally-infected howler monkeys through identification of gametocyte transcripts and estimation of gametocyte density through reverse-transcription quantitative PCR. Consequently, it was possible to identify through molecular methods howler monkeys from the Brazilian Atlantic Forest infected with gametocytes of *P. simium*, which were potentially infective to human beings. This finding can contribute to understanding the occurrence and intensity of malaria transmission from NHP reservoir hosts to mosquito vectors, and so help to define public policies for the control, prevention and eventual elimination of malaria.

## Methods

### Ethical approval

Capture, handling and blood sampling of free-living primate in the municipality of Joinville in the state of Santa Catarina, Brazil, was approved by the Ethical Committee on the Use of Animals of the Fundação Universidade Regional de Blumenau (FURB) under the protocol nº 012/15. The Brazilian government authorized this study, access to, and transport of, biological samples through the Sistema de Autorização e Informação em biodiversidade (SISBIO) no. 43375–4/2015 (for CPRJ samples) and nº 43375–6 (for Joinville samples).

### Non-human primate samples

Whole blood samples were obtained from NHP from fragments of the Brazilian Atlantic Forest located in the states of Santa Catarina and Rio de Janeiro. The sampled animals were free-living NHP from Joinville (n = 32) and captive primates from the Centro de Primatologia do Estado do Rio de Janeiro (CPRJ) (n = 3), comprising two species of the Neotropical primates, the brown howler monkey, *Alouatta guariba clamitans* (the Atelidae family) (n = 33) and the black-headed uakari, *Cacajao melanocephalus* (the Pitheciidae family) (n = 2). The samples used here have been both previously diagnosed and published by our group using conventional PCR of *18S rRNA* locus [32] and PCR–RFLP of *cytochrome* c *oxidase I* locus [33, 34]. The samples chosen for inclusion in this study came from: 11 *P. simium*-infected NHP, five *P. brasilianum*-infected NHP, and 13 NHP with mixed-infections (i. e. both *P. simium* and *P. brasilianum*), as well as six NHP diagnosed as non-infected. Aliquots of whole blood samples were stored in RNAprotect (Qiagen) at a 1:5 ratio and stored at −20 °C until RNA extraction.

### RNA extraction and cDNA synthesis

RNA extraction was performed using different blood in RNAprotect volumes (100 to 1000 μL) using the commercial RNeasy Mini Kit (Qiagen), according to manufacturer’s protocol, which resulted in 30 μL of RNA. DNA removal was done immediately after RNA extraction using the Turbo DNA-free™ Kit (Invitrogen, Life Technologies). In order to synthesize the complementary DNA (cDNA), reverse transcription was performed using the enzyme SuperScript^®^ IV Reverse Transcriptase (SSIV—Invitrogen, Life Technologies) and random primers (Invitrogen, Life Technologies), according to the manufacturer’s instructions. For a final volume of 20 μL, a reverse transcription reaction mix was made containing 1 μL of 50 μM random hexamers, 1 μL of 10 mM dNTPmix, up to 11 μL of template RNA (up to 500 ng mRNA), and up to 13 μL of nuclease-free water. The reaction was incubated at 65 °C for 5 min, and then on ice for at least 1 min. Next, 4 μL of 5 × SSIV Buffer, 1 μL of 100 mM DTT, 1 μL of RNaseOUT™ Recombinant RNase Inhibitor and 1 μL of SSIV were added. The reactions were performed on a Veriti 96-well Thermal Cycler (Thermo Fisher Scientific) at 23 °C for 10 min, 55 °C for 10 min, and then 80 °C for 10 min.

### Amplification of *18S rRNA*, *Pss25* and *Pss48/45*

For confirmation of *Plasmodium* infections and detection of *P. simium* gametocytes, three different quantitative PCR (qPCR) protocols were performed using the cDNA obtained as described above. For the *18S rRNA* transcript, the primers used were those described by Wampfler et al*.* [16]. For the *Pss25* (*P. simium* sexual antigen orthologue to *Pvs25*) transcript, the primers used were designed for *Pvs25* by the same authors, because of the high identity between *P. simium* and *P. vivax* (Additional file 1). For the *Pss48/45* (*P. simium* sexual antigen orthologue to *Pvs48/45*) transcript, new primers were designed using the OligoAnalyzer software based on the sequence available in GenBank (*P. vivax* transmission-blocking target antigen precursor, putative, Accession Number XM_001614196.1). The best of primer sequences identified were 5'-CTCTACCGGAACCATGTTGAAG-3' (forward) and 5'-GACGTACTTGACCTCTCCTTTG-3' (reverse), which generate a fragment of 109 base pairs. For *18S rRNA* transcript amplification, the qPCR reaction was performed using a 10 μL final volume containing 900 nM of each primer, 5 μL GoTaq^®^ qPCR Master Mix, and 1 μL cDNA. The *Pss25* and *Pss48/45* reactions were performed, separately, using 10 μL final volume containing 200 nM of each primer (forward and reverse), 5 μL GoTaq^®^ qPCR Master Mix, and 1 μL cDNA. The qPCR assays were performed on an automatic thermocycler ViiA7 Real-Time PCR System (Thermo Fisher Scientific) with an initial denaturation at 95 °C for 2 min, followed by 40 cycles at 95 °C for 15 s and 60 °C for 1 min. A final cycling for dissociation curve analysis of 95 °C for 15 s, 60 °C for 1 min and 95 °C for 15 s was used. A sample previously diagnosed as *P. vivax* by *18S RNA* PCR described by Snounou et al*.* [32], and positive by RT-qPCR for *18S rRNA*, *Pvs25* and *Pvs48/45* was used as a positive control in all qPCR assays, as well as a negative control (without cDNA). The results of the qPCR were analysed using the QuantStudio Real Time PCR Software v1.3.7.

### RNA quantification and estimation of *P. simium* gametocyte density

Plasmids containing the *18S rRNA* and *Pvs25* fragments of interest were previously prepared by Salazar [35] and were used to perform absolute quantification using a standard curve of *P. simium* gametocytes. Plasmid DNA concentration was obtained by fluorimetric quantification using a Qubit 4 (Invitrogen). The plasmid copy number (PCN) was calculated to determine the dilutions to be used for the standard curve, which was constructed based on seven ten-fold serial dilutions (ranging from 1 × 10^6^ to 1 × 10^1^). The number of gametocytes based on *Pss25* transcript copy number were estimated using a previously published conversion factor (4.17 *Pvs25* transcripts/µL is equal to one gametocyte/µL) calculated by Koepfli et al*.*, which was based on a random-effect model from log10-transformed quantities of gametocyte trend-lines [36]. For the quantification analysis, samples with a quantification cycle (Cq) ≥ 34 were not considered, since a large variation was observed between the replicates above this value (Cq SD > 0.3), resulting in unreliable quantification.

### Statistical analyses

The statistical analyses were performed using GraphPad Prism 8.0.2 (GraphPad Software, San Diego, CA, USA). Linear regression was used to compare the Cq values obtained for *18S rRNA*, *Pss25*, and *Pss48/45*, while non-linear correlation was used to compare the copy number between the *18S rRNA* and *Pss25* transcripts, through the non-parametric Spearman’s rank correlation coefficient. The sample J14 was excluded from the linear correlation analysis. The significance level of 5% was considered for all analysis.

## Results

### Detection of *Plasmodium* infection and *P. simium* gametocytes

Thirty-five samples of NHP previously evaluated for simian malaria were used for detection of *Plasmodium* infection by RT-qPCR of *18S rRNA*, and gametocyte-specific identification through detection of the *Pss25* and *Pss48/45* transcripts (Table 1). The results for *18S rRNA* showed a high percentage of positive samples (74.3%, 26 samples), mostly in agreement with our previous molecular diagnosis (82.9%, 29 of the samples).

**Table 1:** Detection of asexual (*18S rRNA*) and sexual (*Pss25* and *Pss48/45*) transcripts of *Plasmodium* in non-human primates from the Atlantic Forest

Target	*18S rRNA*		*Pss25*		*Pss48/45*	
Previous PCR result	Amplification	Number of samples (percentage)	Amplification	Number of samples (percentage)	Amplification	Number of samples (percentage)
*Ps* + samples (single n = 11 *or mixed n* = *13)*	+	21 (87.5%)	+	13 (62%)	+	7 (54%)
					−	6 (46%)
			−	8 (38%)	−	8 (100%)
	−	3 (12.5%)	−	3 (100%)	−	3 (100%)
*Pbr* + single (n = 5)	+	2 (40%)	+	1 (50%)	−	1 (100%)
			−	1 (50%)	−	1 (100%)
	−	3 (60%)	−	3 (100%)	−	3 (100%)
Negative samples (n = 6)	+	3 (50%)	−	3 (100%)	−	3 (100%)
	−	3 (50%)	−	3 (100%)	−	3 (100%)

The previously published diagnosis is expressed as: positive for *P. simium* (*Ps* +), including both single and mixed *P. simium* infection (with *P. brasilianum*); positive for *P. brasilianum* (*Pbr* +); and parasite-negative samples. For more details about previously published diagnosis please see Alvarenga et al*.* [31] and Nunes et al*.* [32]. The amplification results are expressed as positive ( +) or negative (−) for each transcript. Pss25—*P. simium* sexual antigen 25; Pss48/45—*P. simium* sexual antigen 48/45

Considering only the *P. simium* samples that were positive for the *18S rRNA* transcript (n = 21), 13 samples were also positive for *Pss25*, indicating that 61.9% of the *18S rRNA*-positive samples contained *P. simium* gametocytes, of which seven samples also amplified *Pss48/45* transcripts (i. e. 53.8% of the *Pss25* positive samples) (Table 1). 

Overall, nine samples (25.7%) were negative for all three assayed loci. Another nine samples showed discordant results between our previously molecular diagnosis and the amplification reported here of *18S rRNA* transcripts by qPCR (Table 2, highlighted in orange). Surprisingly, one sample previously diagnosed as positive for *P. brasilianum* gave amplification using the *Pvs25* primers (J21, highlighted in orange in Table 2).

**Table 2:**
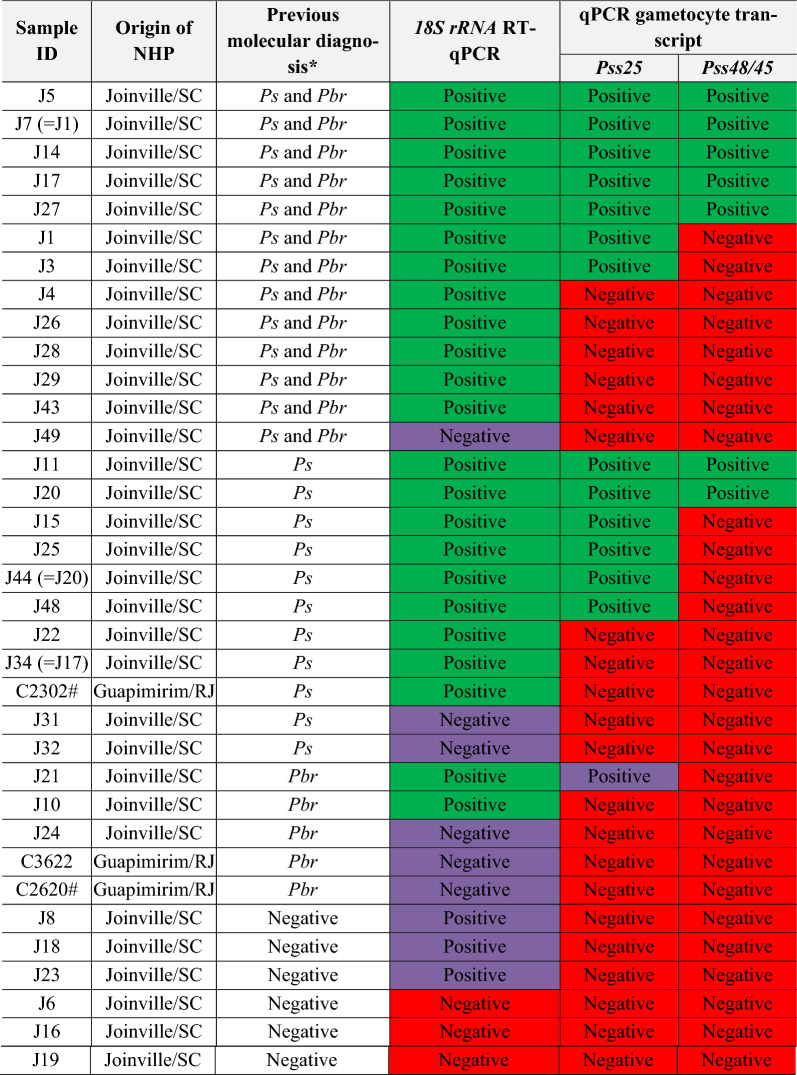
Transcript detection of *Plasmodium 18S rRNA*, *Pss25* and *Pss48/45* in non-human primate samples from the Atlantic Forest

Results of RT-qPCR of *18S rRNA*, *Plasmodium simium* sexual antigen 25 (*Pss25*) and *P. simium* sexual antigen 48/45 (*Pss48/45*) are showed as positive (green cells), negative (red cells) or discordant results comparing to previously published diagnosis (purple cells)

^*^The molecular diagnosis was previously published by our group [31, 32]. The results are expressed as positive to *P. simium* (*Ps*), *P. brasilianum (Pbr)*, mixed infection (*Ps and Pbr*), or negative

^#^All animals are brown howler monkeys (*Alouatta guariba clamitans)*, except C2302 and C2620 which are black-headed uakari (*Cacajao melanocephalus)*. Three samples were collected in different time points from animals previously caught (indicated in parenthesis)

Among all the samples positive for at least one of the three assayed transcripts, the observed Cq values were lowest for *18S rRNA*, with an average of 24.6 (range 13.8 to 33.5), while those observed for *Pss25* were higher with a Cq average of 32.1 (range 24.9 to 38.4) (Table 3). The Pss45/48 target showed a Cq average of 33.5 (range 28.9 to 36.1).

**Table 3:** Cq values and transcript quantification of the *18S rRNA* and *Plasmodium simium* sexual antigen 25 (*Pss25*) targets in non-human primate samples

Sample ID	*18S rRNA* RT-qPCR		*Pss25* RT-qPCR		Number of estimated gametocytes/µL^#^
	Cq	Quantity (copies/µL)	Cq	Quantity (copies/µL)	
J1	24.3	8.76	33.2	0.07	0.02
J3	20.6	141.22	–		
J4	29.5	0.17	–		
J5	16.6	2,912.17	29.3	1.12	0.27
J7	17.0	2,248.03	29.2	1.21	0.29
J8**	32.1	0.03			
J10*	27.9	0.58	–		
J11	13.8	25,170.71	28.0	2.79	0.67
J14	15.1	9,614.21	24.9	24.17	5.80
J15	21.7	65.41	–		
J17	18.5	707.94	30.5	0.48	0.12
J18**	32.6	0.02			
J20	24.1	10.19	33.9	0.05	0.01
J21*	29.6	0.16	–		
J22	23.5	15.98	–		
J23**	32.4	0.02	–		
J25	21.2	91.59	33.7	0.05	0.01
J26	28.2	0.46	–		
J27	17.1	2,065.79	29.8	0.76	0.18
J28	28.0	0.52	–		
J29	33.5	0.01	–		
J34	31.2	0.05	–		
J43	22.7	29.72	–		
J44	20.0	225.41	33.8	0.05	0.01
J48	25.5	3.59	–		
C2302	31.6	0.04	–		

All samples were previously detected as positive for *P. simium* or mixed infection, except for the indicated samples infected by *P. brasilianum* (*) or not infected (**). Only samples with Cq < 34 were included for quantification

^#^Number of gametocytes estimated according to Koepfli et al*.* [53]: 1 gametocyte = 4.17 *Pss25* transcripts

**Fig. 1:**
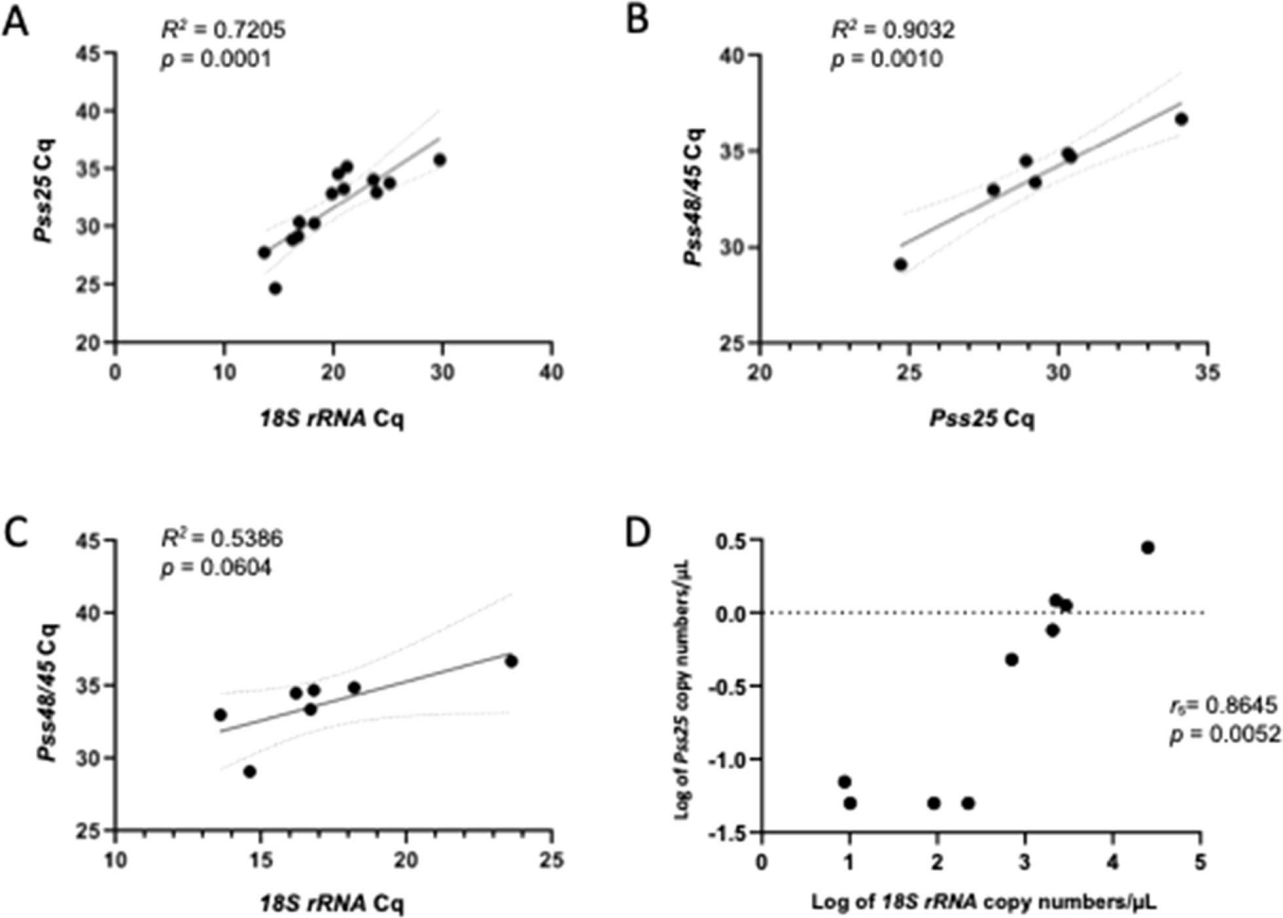
Correlation analysis of Cq values and copy number of *18S rRNA* and gametocyte-specific transcripts. The linear regression of Cq values between *18S rRNA* and *Pss25* (**A**), *Pss25* and *Pss48/45* transcripts (**B**), and between *18S rRNA* and *Pss48/45* (**C**). Dashed lines indicate 95% confidence interval. A non-linear correlation between estimated copy number of *18S rRNA* and *Pss25* transcripts using non-linear Spearman’s rank correlation coefficient (**D**)

A strong positive linear correlation was identified between the Cq values for *18S rRNA* and *Pss25* (R^2^ = 0.7205, *P* = 0.0001) (Fig. 1A), and between those for *Pss25* and *Pss48/45* (R^2^ = 0.9032, *P* = 0.0010) (Fig. 1B). However, a significant linear correlation was not observed between the Cq values for *18S rRNA* and *Pss48/45* (R^2^ = 0.5386, *P* = 0.0604) (Fig. 1C).

### Quantification of *P. simium* gametocytes

Absolute quantification was performed using standard curves to estimate the copy number of both *18S rRNA* and *Pss25*. Quantification was not performed for *Pss48/45*, because amplification of this locus by qPCR gave larger variation, which directly interfered in the accuracy of quantification. The twenty-six RNA samples from the *18S rRNA*-positive NHP gave estimated transcript copy numbers ranging from 0.01 to 25,170.71 copies/µL, with an average of 1665.88 copies/µL (Table 3). For *Pss25* quantification, the amount of transcript ranged from 0.05 to 24.17 copies/µL, with an average of 3.07 copies/µL (Table 3). However, since only samples with Cq < 34 were considered in the analysis, gametocyte quantification was assessed for only ten samples (with the remaining three *Pvs25*-positive samples excluded). The non-linear Spearman's rank correlation coefficient revealed a positive correlation between the copy numbers of *Pvs25* and *18S rRNA* (r_s_ = 0.8645, *P* = 0.0052, log-transformed values) (Fig. 1D). The estimated number of gametocytes per microlitre (calculated from *Pss25* transcript copy number) was very low (less than 1 gametocyte/µL), except for one animal (J14) which showed a high density of gametocytes (5.8 gametocytes/µL) (Table 3).

## Discussion

In Brazil, the highest prevalence of malaria cases is in the Amazon region, with over 99% of the national notifications. However, autochthonous infections have also been described in the extra-Amazonian region, mainly in Atlantic Forest areas. The state of Rio de Janeiro recorded an average of four autochthonous malaria cases per year from 2006 to 2014 [37]. However, in 2015 and 2016, these rates increased to 33 and 16, respectively. Mitochondrial genome analyses of the parasites infecting non-human primates and humans from this outbreak revealed that they were *P. simium*, validating that malaria has a zoonotic transmission in this region [12, 33]. Brown howler monkeys (*Alouatta g. clamitans*) have been suggested to be the main reservoir of malaria in Atlantic Forest areas. In the current study, more than 60% of *Plasmodium*-positive samples from this non-human primate species were positive for molecular markers consistent with the presence of gametocytes in their blood, demonstrating the potential of this vertebrate host species to act as a reservoir for malaria transmission. The failure to detect gametocytes in all *P. simium*-infected howler monkeys may be because the low densities of the gametocytes in the peripheral blood or because of their sequestration in the bone narrow, as showed for *P. vivax* gametocytes [38, 39]. Interestingly, one howler monkey, previously diagnosed as positive for *P. brasilianum*, had positive amplification using the primers to *Pvs25* locus. This amplification was unexpected because of the many polymorphisms within the primers and probe binding sites in the *Pvs25* orthologue of *P. brasilianum* (Additional file 1). This animal might have had a mixed infection, common in the region it was from [34], with low levels of *P. simium*. In addition, related to the other NHP species evaluated here, one black-headed uakari (*Cacajao melanochephalus*) was positive for *P. simium* infection, but negative for both gametocyte transcripts. Since this species is an endemic of the Amazon, these results suggest that this monkey species might be a dead-end host for *P. simium*, without epidemiological importance for malaria transmission in the Atlantic Forest. However, because this animal was housed in captivity in the Atlantic Forest, it was not possible to exclude that the gametocytes were in low densities. This requires further investigation, since only two specimens of the black-headed uakari were included here, and large sample sizes are needed.

A deeper understanding of gametocyte carriers is essential to better understand their potential for malaria transmission. There are only a few studies of identifying gametocytes from *P. simium* and *P. brasilianum*, all based on the detection of this stage through blood smears under an optical microscope [12, 40, 41]. Molecular identification was not performed for these two *Plasmodium* species, since gametocyte genes were not previously studied, and their sequences were only recently elucidated with the sequencing of the complete genomes of these two parasites [42–44]. Considering the high similarity among *P. simium* and *P. vivax* (Additional file 1), the studies about gametocytes detection from the later were used here. Some studies have shown that *P. vivax* parasitaemia and gametocytaemia are tightly linked [36, 45]. Moreover, infectivity for mosquitoes is known to be positively associated with gametocyte density, with variations between mosquito species and study areas [45–50]. In Ethiopia, Golassa et al*.* [51] showed that 86.4% of asymptomatic *P. vivax* cases had asexual parasites and 13.6% had both asexual and gametocytes, another study estimated the contribution of asymptomatic for mosquito infections in 79 to 92% [48]. Kosasih et al*.* [52] showed that the prevalence of gametocytes is higher in individuals with microscopic than sub-microscopic *P. vivax* infections (92% versus 26%). Imwong et al*.* [53] used an ultrasensitive PCR (uPCR) method to identify many individuals infected with *P. vivax* in malaria endemic areas and demonstrated that parasitaemia persists in humans at levels that optimize the probability of generating densities of transmissible gametocytes without causing illness. The authors reported that as gametocytes are eliminated more slowly than asexual stages from the peripheral blood, a significant proportion of the parasites detected by uPCR in asymptomatic individuals are likely to be gametocytes. Additionally, a recent study by Almeida et al*.* [54] using human samples with very low parasitaemia showed that there is a possibility of mosquito infection. The authors demonstrated, through an artificial feeding assay, that blood from asymptomatic individuals can act as a source of *P. vivax* transmission to the vector *Anopheles (Nyssorhynchus) darlingi*, the main vector of *P. vivax* in the Brazilian Amazon. Although lower infectivity rates were observed for blood from asymptomatic individuals (2.5%) compared to symptomatic patients (43.4%), many asymptomatic carriers maintained parasitaemia for several weeks, indicating their potential role as an infectious reservoir. Symptomatic *P. vivax* infected individuals are able to infect mosquitoes at variable rates from 20–90% [48, 49, 55–57] and asymptomatic individuals can also be infective, but at much lower rates [48, 49, 57]. Although lower gametocyte densities in asymptomatic individuals are obviously much less infectious, they can contribute significantly to transmission by having a higher frequency (> = 80%) in the population [58–61]. Therefore, in Amazonia sub-microscopic and asymptomatic *P. vivax* infections constitute the main infectious reservoir of this parasite [62].

Molecular methods have been developed to detect transcripts of gametocyte-specific genes, with a detection limit of 0.02–10 gametocytes per microlitre of blood [63, 64]. According to Bharti et al*.* [56], the number of *Pvs25* transcripts correlates positively with the number of circulating mature gametocytes and can be used as an indirect estimate of gametocyte density in the sample. Koepfli et al*.* [36] has gone further, calculating a conversion factor for *Pvs25* transcripts into the number of gametocytes. Applying this conversion factor, which is defined for *P. vivax*, a high frequency of low gametocyte densities—less than one gametocyte/μL—was observed here. Therefore, as suggested for *P. vivax* human infections, the low densities of gametocytes could be compensated by the high frequency of infection. Nonetheless, one howler monkey showed higher levels of gametocytes, which may act as a potential “super-spreader” of *P. simium* infection. Historical studies have suggested around 10 gametocytes/μL is an infective density [65, 66]. Recently, two studies confirmed that gametocytes as few as 1 gametocyte/μL were able to infect *Anopheles dirus* and *Anopheles stephensi* [45, 67]. In the Atlantic Forest, *Anopheles* from *Kerteszia* group has been incriminated as the main vector of malaria [9, 68]. High densities of these mosquitoes species, and their highly voracious blood-feeding habits [69], together with their requirement to have more than one blood meal in order to complete their gonothrophic cycle [68], may all increase the chances of their transmitting malaria. However, functional assays, such as skin feeding assays (SFA) or direct membrane feeding assays (DMFA) using different densities of gametocytes remain to be performed in order to identify the infective density of gametocytes required for *P. simium* transmission to mosquitoes. Previously, this density could not be identified, since the experimental infections performed using laboratory *P. simium* infection of monkeys only quantified infective gametocytes by microscopy and did not perform mosquito infections using a range of different gametocyte densities [70].

Interruption of malaria transmission is considered a priority task in the process of malaria elimination [71, 72]. Therefore, it is extremely important to understand the epidemiology of gametocytes and the contribution of asymptomatic and sub-microscopic carriers acting as reservoirs, especially in low transmission settings. In this context, high frequencies of infected howler monkeys, can carry gametocytes, even with very low densities, potentially contributing to the infection of mosquitoes and, consequently, human beings. The data presented here could help in the mathematical modelling of the dynamics of zoonotic malaria transmission, which may consider the individual variation in the levels of gametocytes among reservoirs, with a high frequency of howler monkeys with low levels of gametocytes and a few NHP with higher levels. Moreover, these models must take into account the presence of other non-human primate species which maybe a dead-end hosts. The modellers have also to consider that the distribution of gametocyte between different individual hosts within a single reservoir host population/species is expected exponential. However, more studies need to be done to assess mosquito infection rates and to help to understand to what extent NHP can act as reservoir hosts and contribute to the maintenance of the *Plasmodium* life cycle in the Atlantic Forest.

## Conclusion

Gametocytes were detected in *P. simium* infected brown howler monkeys (*Alouatta g. clamitans*). This is strong evidence that howler monkeys are acting as *Plasmodium* reservoirs in the Atlantic Forest. Transmission could be maintained by the high frequency of low-level gametocyte carriers and the low frequency of high-level carriers. This finding will contribute towards the modelling of zoonotic malaria transmission and definition of public policies for malaria control, prevention and elimination.

## Supplementary Information

**Additional file 1:** Alignment of *Pvs25* gene sequence and its orthologous from *Plasmodium simium*, *P. malariae* and *P. brasilianum*.

## Data Availability

The authors confirm that all data reported in the manuscript are publicly available.

## Abbreviations

*18S** rRNA*: Small subunit 18S of ribosomal RNA gene
*Pvs25*: *Plasmodium vivax* Ookinete surface protein gene
*Pss25*: *Plasmodium simium* Sexual antigen gene orthologue to *Pvs25*
*Pvs48/45*: *Plasmodium vivax* Gametocyte antigen gene
*Pss48/45*: *Plasmodium simium* Sexual antigen gene orthologue to *Pvs48/45*
qPCR: Quantitative real-time PCR
RT-qPCR: Reverse transcription quantitative real-time PCR
gDNA: Genomic DNA
cDNA: Complementary DNA
PCN: Plasmid copy number
NHP: Non-human primates
*Ps*: *Plasmodium simium*
*Pbr*: *Plasmodium brasilianum*
Neg: Negative
Cq: Quantification cycle
uPCR: Ultrasensitive PCR

## References

[1] WHO (2023). World malaria report 2022.

[2] Ministério da Saúde/Secretaria de Vigilância em Saúde. Malária 2021. Boletim Epidemiológico. Número especial. Brasília: MS/SVS; 2021. 100 pp.

[3] Melo JO, Padilha MAO, Barbosa RTA, Alonso WJ, Vittor AY, Laporta GZ (2020). Evaluation of the malaria elimination policy in Brazil: a systematic review and epidemiological analysis study. Trop Biomed.

[4] Singh B, Kim Sung L, Matusop A, Radhakrishnan A, Shamsul SS, Cox-Singh J (2004). A large focus of naturally acquired *Plasmodium knowlesi* infections in human beings. Lancet.

[5] Cox-Singh J, Davis TM, Lee KS, Shamsul SS, Matusop A, Ratnam S (2008). *Plasmodium knowlesi* malaria in humans is widely distributed and potentially life threatening. Clin Infect Dis.

[6] Singh B, Daneshvar C (2013). Human infections and detection of *Plasmodium knowlesi*. Clin Microbiol Rev.

[7] Yusof R, Lau YL, Mahmud R, Fong MY, Jelip J, Ngian HU (2014). High proportion of knowlesi malaria in recent malaria cases in Malaysia. Malar J.

[8] Ta TH, Hisam S, Lanza M, Jiram AI, Ismail N, Rubio JM (2014). First case of a naturally acquired human infection with *Plasmodium cynomolgi*. Malar J.

[9] Deane LM, Deane MP, Ferreira NJ (1966). Studies on transmission of simian malaria and on the natural infection of man with *Plasmodium simium* in Brazil. Bull World Health Organ.

[10] Arruda ME, Nardini EH, Nussenzweig RS, Cchrane AH (1989). Sero-epidemiological studies of malaria in indian tribes and monkeys of the Amazon basin of Brazil. Am J Trop Med Hyg.

[11] Lalremruata A, Magris M, Vivas-Martínez S, Koehler M, Esen M, Kempaiah P (2015). Natural infection of *Plasmodium brasilianum* in humans: man and monkey share quartan malaria parasites in the Venezuelan Amazon. EBioMedicine.

[12] Brasil P, Zalis MG, Pina-Costa A, Siqueira AM, Bianco C, Silva S (2017). Outbreak of human malaria caused by *Plasmodium simium* in the Atlantic forest in Rio de Janeiro: a molecular epidemiological investigation. Lancet Glob Health.

[13] de Alvarenga DAM, de Pina-Costa A, de Sousa TN, Pissinatti A, Zalis MG, Suaréz-Mutis MC (2015). Simian malaria in the Brazilian Atlantic forest: first description of natural infection of capuchin monkeys (Cebinae subfamily) by *Plasmodium simium*. Malar J.

[14] Abreu FVS, Santos ED, Mello ARL, Gomes LR, Alvarenga DAM, Gomes MQ (2019). Howler monkeys are the reservoir of malarial parasites causing zoonotic infections in the Atlantic forest of Rio de Janeiro. PLoS Negl Trop Dis.

[15] Duarte AM, Malafronte Rdos S, Cerutti C, Curado I, de Paiva BR, Maeda AY (2008). Natural *Plasmodium* infections in Brazilian wild monkeys: reservoirs for human infections?. Acta Trop.

[16] Wampfler R, Mwingira F, Javati S, Robinson L, Betuela I, Siba P (2013). Strategies for detection of *Plasmodium* species gametocytes. PLoS ONE.

[17] Lover AA, Baird JK, Gosling R, Price RN (2018). Malaria elimination: time to target all species. Am J Trop Med Hyg.

[18] Carter R, Mendis KN, Miller LH, Molineaux L, Saul A (2000). Malaria transmission-blocking vaccines—how can their development be supported?. Nat Med.

[19] Ouédraogo AL, Bousema T, Schneider P, de Vlas SJ, Ilboudo-Sanogo E, Cuzin-Ouattara N (2009). Substantial contribution of submicroscopical *Plasmodium falciparum* gametocyte carriage to the infectious reservoir in an area of seasonal transmission. PLoS ONE.

[20] Churcher TS, Bousema T, Walker M, Drakeley C, Schneider P, Ouédraogo AL (2013). Predicting mosquito infection from *Plasmodium falciparum* gametocyte density and estimating the reservoir of infection. Elife.

[21] Bantuchai S, Imad H, Nguitragool W (2022). *Plasmodium vivax* gametocytes and transmission. Parasitol Int.

[22] Baton LA, Ranford-Cartwright LC (2005). Spreading the seeds of million-murdering death: metamorphoses of malaria in the mosquito. Trends Parasitol.

[23] Chaves LB, Perce-da-Silva DS, Totino PRR, Riccio EKP, Baptista BO, de Souza ABL (2019). *Plasmodium vivax* ookinete surface protein (Pvs25) is highly conserved among field isolates from five different regions of the Brazilian Amazon. Infect Genet Evol.

[24] Duffy PE (2021). Transmission-blocking vaccines: harnessing herd immunity for malaria elimination. Expert Rev Vaccines.

[25] Tomas AM, Margos G, Dimopoulos G, van Lin LH, de Koning-Ward TF, Sinha R (2001). P25 and P28 proteins of the malaria ookinete surface have multiple and partially redundant functions. EMBO J.

[26] Vermeulen AN, Ponnudurai T, Beckers PJ, Verhave JP, Smits MA, Meuwissen JH (1985). Sequential expression of antigens on sexual stages of *Plasmodium falciparum* accessible to transmission-blocking antibodies in the mosquito. J Exp Med.

[27] Kumar N (1987). Target antigens of malaria transmission blocking immunity exist as a stable membrane bound complex. Parasite Immunol.

[28] Kocken CH, Jansen J, Kaan AM, Beckers PJ, Ponnudurai T, Kaslow DC, Konings RN, Schoenmakers JG (1993). Cloning and expression of the gene coding for the transmission blocking target antigen Pfs48/45 of *Plasmodium falciparum*. Mol Biochem Parasitol.

[29] van Dijk MR, Janse CJ, Thompson J, Waters AP, Braks JA, Dodemont HJ, Stunnenberg HG, van Gemert GJ, Sauerwein RW, Eling W (2001). A central role for P48/45 in malaria parasite male gamete fertility. Cell.

[30] Feng H, Gupta B, Wang M, Zheng W, Zheng L, Zhu X (2015). Genetic diversity of transmission-blocking vaccine candidate Pvs48/45 in *Plasmodium vivax* populations in China. Parasit Vectors.

[31] Kim A, Popovici J, Menard D, Serre D (2019). *Plasmodium vivax* transcriptomes reveal stage-specific chloroquine response and differential regulation of male and female gametocytes. Nat Commun.

[32] Snounou G, Viriyakosol S, Zhu XP, Jarra W, Pinheiro L, do Rosario VE, Thaithong S, Brown KN (1993). High sensitivity of detection of human malaria parasites by the use of nested polymerase chain reaction. Mol Biochem Parasitol.

[33] de Alvarenga DAM, Culleton R, de Pina-Costa A, Rodrigues DF, Bianco C, Silva S (2018). An assay for the identification of *Plasmodium simium* infection for diagnosis of zoonotic malaria in the Brazilian Atlantic forest. Sci Rep.

[34] Nunes AJD, Alvarenga DAM, de Souza Junior JC, Peruchi AR, Gonçalves GHP, Hirano ZMB (2020). *Plasmodium* infection and its association with biochemical and haematological parameters in free-living *Alouatta guariba clamitans* (Cabrera, 1940) (Primates: Atelidae) in Southern Brazil. Mem Inst Oswaldo Cruz.

[35] Salazar, YEAR. Resposta terapêutica na malária por *Plasmodium vivax*: variabilidade genética de enzimas metabolizadoras da primaquina e o clearance de gametócitos. Belo Horizonte: s.n, 2022. 85 p. Dissertação-Ministério da Saúde. Fundação Oswaldo Cruz. Instituto René Rachou. Programa de Pós-Graduação em Ciências da Saúde. https://www.arca.fiocruz.br/bitstream/handle/icict/55234/D_22_Yanka%20Salazar.pdf?sequence=2&isAllowed=y. Accessed 08 Nov 2022.

[36] Koepfli C, Robinson LJ, Rarau P, Salib M, Sambale N, Wampfler R (2015). Blood-stage parasitaemia and age determine Plasmodium falciparum and P vivax gametocytaemia in Papua New Guinea. PLoS One.

[37] Miguel RB, Peiter PC, de Albuquerque H, Coura JR, Moza PG, Pina Costa A (2014). Malaria in the state of Rio de Janeiro, Brazil, an Atlantic forest area: an assessment using the health surveillance service. Mem Inst Oswaldo Cruz.

[38] Obaldia N, Meibalan E, Sa JM, Ma S, Clark MA, Mejia P (2018). Bone marrow is a major parasite reservoir in *Plasmodium vivax* infection. mBio.

[39] Salazar Alvarez LC, Vera Lizcano O, da Silva Barros DKA, Baia-da-Silva DC, Monteiro WM, Pimenta PFP (2021). *Plasmodium vivax* gametocytes adherence to bone marrow endothelial cells. Front Cell Infect Microbiol.

[40] Taliaferro WH, Taliaferro LG (1934). Morphology, periodicity and course of infection of *Plasmodium brasilianum* in Panamanian monkeys. Am J Epidemiol.

[41] Da Fonseca F (1951). Plasmódio de primata do Brasil. Mem Inst Oswaldo Cruz.

[42] Bajic M, Ravishankar S, Sheth M, Rowe LA, Pacheco MA, Patel DS (2022). The first complete genome of the simian malaria parasite *Plasmodium brasilianum*. Sci Rep.

[43] Mourier T, de Alvarenga DAM, Kaushik A, de Pina-Costa A, Douvropoulou O, Guan Q (2021). The genome of the zoonotic malaria parasite *Plasmodium simium* reveals adaptations to host switching. BMC Biol.

[44] de Oliveira TC, Rodrigues PT, Early AM, Duarte AMRC, Buery JC, Bueno MG (2021). *Plasmodium simium*: population genomics reveals the origin of a reverse zoonosis. J Infect Dis.

[45] Kiattibutr K, Roobsoong W, Sriwichai P, Saeseu T, Rachaphaew N, Suansomjit C (2017). Infectivity of symptomatic and asymptomatic *Plasmodium vivax* infections to a Southeast Asian vector. Anopheles dirus Int J Parasitol.

[46] Lindblade KA, Steinhardt L, Samuels A, Kachur SP, Slutsker L (2013). The silent threat: asymptomatic parasitemia and malaria transmission. Expert Rev Anti Infect Ther.

[47] Lin JT, Ubalee R, Lon C, Balasubramanian S, Kuntawunginn W, Rahman R (2016). Microscopic *Plasmodium falciparum* gametocytemia and infectivity to mosquitoes in Cambodia. J Infect Dis.

[48] Tadesse FG, Slater HC, Chali W, Teelen K, Lanke K, Belachew M (2018). The relative contribution of symptomatic and asymptomatic *Plasmodium vivax* and *Plasmodium falciparum* infections to the infectious reservoir in a low-endemic setting in Ethiopia. Clin Infect Dis.

[49] Martins-Campos KM, Kuehn A, Almeida A, Duarte APM, Sampaio VS, Rodriguez ÍC (2018). Infection of *Anopheles aquasalis* from symptomatic and asymptomatic *Plasmodium vivax* infections in Manaus, western Brazilian Amazon. Parasit Vectors.

[50] Slater HC, Ross A, Felger I, Hofmann NE, Robinson L, Cook J (2019). The temporal dynamics and infectiousness of subpatent *Plasmodium falciparum* infections in relation to parasite density. Nat Commun.

[51] Golassa L, Baliraine FN, Enweji N, Erko B, Swedberg G, Aseffa A (2015). Microscopic and molecular evidence of the presence of asymptomatic *Plasmodium falciparum* and *Plasmodium vivax* infections in an area with low, seasonal and unstable malaria transmission in Ethiopia. BMC Infect Dis.

[52] Kosasih A, Koepfli C, Dahlan MS, Hawley WA, Baird JK, Mueller I (2021). Gametocyte carriage of *Plasmodium falciparum* (pfs25) and *Plasmodium vivax* (pvs25) during mass screening and treatment in West Timor, Indonesia: a longitudinal prospective study. Malar J.

[53] Imwong M, Nguyen TN, Tripura R, Peto TJ, Lee SJ, Lwin KM (2015). The epidemiology of subclinical malaria infections in South-East Asia: findings from cross-sectional surveys in Thailand-Myanmar border areas, Cambodia, and Vietnam. Malar J.

[54] Almeida GG, Costa PAC, Araujo MDS, Gomes GR, Carvalho AF, Figueiredo MM (2021). Asymptomatic *Plasmodium vivax* malaria in the Brazilian Amazon: submicroscopic parasitemic blood infects *Nyssorhynchus darlingi*. PLoS Negl Trop Dis.

[55] Sattabongkot J, Maneechai N, Rosenberg R (1991). *Plasmodium vivax*: gametocyte infectivity of naturally infected Thai adults. Parasitology.

[56] Bharti AR, Chuquiyauri R, Brouwer KC, Stancil J, Lin J, Llanos-Cuentas A (2006). Experimental infection of the neotropical malaria vector *Anopheles darlingi* by human patient-derived *Plasmodium vivax* in the Peruvian Amazon. Am J Trop Med Hyg.

[57] Moreno M, Tong C, Guzmán M, Chuquiyauri R, Llanos-Cuentas A, Rodriguez H (2014). Infection of laboratory-colonized *Anopheles darlingi* mosquitoes by *Plasmodium vivax*. Am J Trop Med Hyg.

[58] Zaw MT, Thant M, Hlaing TM, Aung NZ, Thu M, Phumchuea K (2017). Asymptomatic and sub-microscopic malaria infection in Kayah State, eastern Myanmar. Malar J.

[59] Waltmann A, Darcy AW, Harris I, Koepfli C, Lodo J, Vahi V (2015). High rates of asymptomatic, sub-microscopic *Plasmodium vivax* infection and disappearing *Plasmodium falciparum* malaria in an area of low transmission in Solomon islands. PLoS Negl Trop Dis.

[60] Vasquez-Jimenez JM, Arevalo-Herrera M, Henao-Giraldo J, Molina-Gomez K, Arce-Plata M, Vallejo AF (2016). Consistent prevalence of asymptomatic infections in malaria endemic populations in Colombia over time. Malar J.

[61] Nguitragool W, Mueller I, Kumpitak C, Saeseu T, Bantuchai S, Yorsaeng R (2017). Very high carriage of gametocytes in asymptomatic low-density *Plasmodium falciparum* and *P. vivax* infections in western Thailand. Parasit Vectors..

[62] Ferreira MU, Corder RM, Johansen IC, Kattenberg JH, Moreno M, Rosas-Aguirre A (2022). Relative contribution of low-density and asymptomatic infections to *Plasmodium vivax* transmission in the Amazon: pooled analysis of individual participant data from population-based cross-sectional surveys. Lancet Reg Health Am.

[63] Babiker HA, Schneider P, Reece SE (2008). Gametocytes: insights gained during a decade of molecular monitoring. Trends Parasitol.

[64] Lima NF, Bastos MS, Ferreira MU (2012). *Plasmodium vivax*: reverse transcriptase real-time PCR for gametocyte detection and quantitation in clinical samples. Exp Parasitol.

[65] Boyd MF, Kitchen S (1937). On the infectiousness of patients infected with *Plasmodium vivax* and *Plasmodium falciparum1*. Am J Trop Med Hyg.

[66] Jeffery GM (1952). The infection of mosquitoes by *Plasmodium vivax* (Chesson strain) during the early primary parasitemias. Am J Trop Med Hyg.

[67] Collins KA, Wang CY, Adams M, Mitchell H, Robinson GJ, Rampton M (2020). A *Plasmodium vivax* experimental human infection model for evaluating efficacy of interventions. J Clin Invest.

[68] Marrelli MT, Malafronte RS, Sallum MA, Natal D (2007). *Kerteszia* subgenus of *Anopheles* associated with the Brazilian Atlantic rainforest: current knowledge and future challenges. Malar J.

[69] Branquinho MS, Marrelli MT, Curado I, Natal D, Barata JM, Tubaki R (1997). Infection of *Anopheles (Kerteszia) cruzii* by *Plasmodium vivax* and *Plasmodium vivax* variant VK247 in the municipalities of São Vicente and Juquitiba. São Paulo Rev Panam Salud Publica.

[70] Collins WE, Contacos PG, Guinn EG, Skinner JC (1973). *Plasmodium simium* in the *Aotus trivirgatus* monkey. J Parasitol.

[71] Alonso PL, Brown G, Arevalo-Herrera M, Binka F, Chitnis C, Collins F (2011). A research agenda to underpin malaria eradication. PLoS Med.

[72] Ferreira MU, Castro MC (2016). Challenges for malaria elimination in Brazil. Malar J.

