# Supplementary material for: Detection of Plasmodium simium gametocytes in non-human primates from the Brazilian Atlantic Forest
Source: Malar J. 2023 Jun 2;22:170. doi: 10.1186/s12936-023-04601-7 (PMC10239093; doi:10.1186/s12936-023-04601-7)
Supplement: Supplementary file 1 — Additional file 1: Alignment of Pvs25 gene sequence and its orthologous from Plasmodium simium, P. malariae and P. brasilianum. [file 12936_2023_4601_MOESM1_ESM.rtf]

Additional file 1. Alignment of Pvs25 gene sequence and its orthologous from Plasmodium simium, 
P. malariae and P. brasilianum
                        10        20        30        40        50        60        70        80               
               ....|....|....|....|....|....|....|....|....|....|....|....|....|....|....|....|
P.vivax        ATGAACTCCTACTACAGCCTCTTCGTTTTTTTCCTCGTCCAAATTGCGCTAAAGTATAGCAAGGCAGCCGTCACGGTAGA 
P.simium       ................................................................................ 
P.malariae     .....TA....T.....TGCGA.TC....C..TT.AA.G...C....AA....AC...AT..A...AAG.....CA.... 
P.brasilianum  .....TA....T.....TGCGA.TC....C..TT.AA.G...C....AA....AC...AT..A...AAG.....CA.... 

                        90       100       110       120       130       140       150       160        
               ....|....|....|....|....|....|....|....|....|....|....|....|....|....|....|....|
P.vivax        CACCATATGCAAAAATGGACAGCTGGTTCAAATGAGTAACCACTTTAAGTGTATGTGTAACGAAGGGCTGGTGCACCTTT 
P.simium       .........................................................................-...... 
P.malariae     ...GG....T........GT.T...A..........C..T..T...G.....G.A...G.T..T..A.AT..A..TG.AA 
P.brasilianum  ...GG....T........GT.T...A..........C..T..T...G.....G.A...G.T..T..A.AT..A..TG.AA 

                       170       180       190       200       210       220       230       240       
               ....|....|....|....|....|....|....|....|....|....|....|....|....|....|....|....|
P.vivax        CCGAAAATACATGTGAAGAAAAAAATGAATGCAAGAAAGAAACCCTAGGCAAAGCATGCGGGGAATTTGGCCAGTGTATA 
P.simium       ................................................................................ 
P.malariae     AG..CG..GTG......C.G...C.A........AG...G...TAA.TCT...C.T..T.CT..T...TCTACA...G.- 
P.brasilianum  AG..CG..GTG......C.G...C.A........AG...G...TAA.TCT...C.T..T.CT..T...TCTACA...G.- 

                       250       260       270       280       290       300       310       320       
               ....|....|....|....|....|....|....|....|....|....|....|....|....|....|....|....|
P.vivax        GAAAACCCAGACCCAGCACAGGTAAACATGTACAAATGTGGTTGCATTGAGGGCTACACTTTGAAGGAAGACACTTGTGT 
P.simium       ..................A.....................A....................................... 
P.malariae     -----TTTG.--.T.A...-.CC...T.AA..T.CT...ATG..TGA..TA..A..T...AATGTAA....TGT...... 
P.brasilianum  -----TTTG.--.T.A...-.CC...T.AA..T.CT...ATG..TGA..TA..A..T...AATGTAA....TGT...... 

                       330       340       350       360       370       380       390       400       
               ....|....|....|....|....|....|....|....|....|....|....|....|....|....|....|....|
P.vivax        GCTTGATGTATGTCAATACAAAAATTGTGGAGAAAGTGGCGAATGCATTGTTGAGT-ACCTCTCGGAAAC--CAAAAGTG 
P.simium       ........................................................-.....A.......--.C...... 
P.malariae     T.CGTC.......A..A.TGT.TC.....ATA..---..AA....T...T.A..CCC.AA.AAT.A.G.TGTT....C.. 
P.brasilianum  T.CGTC.......A..A.TGT.TC.....ATA..---..AA....T...T.A..CCC.AA.AAT.A.G.TGTT....C.. 

                       410       420       430       440       450       460       470       480       
               ....|....|....|....|....|....|....|....|....|....|....|....|....|....|....|....|
P.vivax        CAGGTTGCTCATGTGCTATTGGCAAAGTCCCCAATCCAGAAGATGAGAAAAAATGTACCAAAACGGGAGAAACTGCTTGT 
P.simium       ................................................................................ 
P.malariae     .CATA..T..T....A...A........T..AG.....A.TA.CA.A..C.TG.....A...GAT..G......AAA... 
P.brasilianum  .CATA..T..T....A...A........T..AG.....A.TA.CA.A..C.TG.....A...GAT..G......AAA... 

                       490       500       510       520       530       540       550       560       
               ....|....|....|....|....|....|....|....|....|....|....|....|....|....|....|....|
P.vivax        CAATTGAAATGTAACACAGATAATGAAGTCTGCAAAAATGTTGAAGGAGTTTACAAGTGCCAGTGTATGGAAGGCTTTAC 
P.simium       ................................................................................ 
P.malariae     AC...A.....CTTA.AGAGC..C...ACT......GT...A......CG...T..A..TG.C...GAA..T..T...T. 
P.brasilianum  AC...A.....CTTA.AGAGC..C...ACT......GT...A......CG...T..A..TG.C...GAA..T..T...T. 

                       570       580       590       600       610       620       630       640       
               ....|....|....|....|....|....|....|....|....|....|....|....|....|....|....|....|
P.vivax        GTTCGACAAAGAGAAAAATGTATGCCTTTCCTATTCTGTATTTAACATCCTAAACTACTCCCTCTTCTTTATCATCCTGC 
P.simium       ................................................................................ 
P.malariae     T..T..T.....AG..GG.A....TAC.G.......A...........TG....T.TAAGTA..A.T..C..T..AGCTT 
P.brasilianum  T..T..T.....AG..GG.A....TAC.G.......A...........TG....T.TAAGTA..A.T..C..T..AGCTT 

                       650       660 
               ....|....|....|....|...
P.vivax        TTGTCCTTTCGTACGTCATATAA 
P.simium       ....................... 
P.malariae     .AAC.TA..TA..TA.T..T.G. 
P.brasilianum  .AAC.TA..TA..TA.T..T.G. 

Accession numbers of sequences from P. vivax – PVP01_0616100; P. simium – sequence recovered through blast from reads SRX10112341; P. malariae - PmUG01_10042200; and P. brasilianum - MKS88_003372. Sequences were recovered from PlasmoDB (Aurrecoechea et al., 2009) and NCBI. Alignment performed using Clustal W at Bioedit platform. Conserved nucleotides are shown as dots. Black squares identify primers and probe binding sites.

Reference
Aurrecoechea C, Brestelli J, Brunk BP, Dommer J, Fischer S, Gajria B, Gao X, Gingle A, Grant G, Harb OS, Heiges M, Innamorato F, Iodice J, Kissinger JC, Kraemer E, Li W, Miller JA, Nayak V, Pennington C, Pinney DF, Roos DS, Ross C, Stoeckert CJ, Jr, Treatman C, Wang H. PlasmoDB: a functional genomic database for malaria parasites. Nucl Acids Res. 2009;37:D539–D543.
